# A randomized pilot trial assessing the reduction of gout episodes in hyperuricemic patients by oral administration of *Ligilactobacillus salivarius* CECT 30632, a strain with the ability to degrade purines

**DOI:** 10.3389/fmicb.2023.1111652

**Published:** 2023-02-14

**Authors:** Juan M. Rodríguez, Marco Garranzo, José Segura, Belén Orgaz, Rebeca Arroyo, Claudio Alba, David Beltrán, Leónides Fernández

**Affiliations:** ^1^Department of Nutrition and Food Science, Complutense University of Madrid, Madrid, Spain; ^2^Department of Galenic Pharmacy and Food Technology, Complutense University of Madrid, Madrid, Spain; ^3^Centro de Diagnóstico Médico, Ayuntamiento de Madrid, Madrid, Spain

**Keywords:** probiotics, *Ligilactobacillus salivarius*, gout, hyperuricemia, uric acid, inosine, guanosine

## Abstract

**Introduction:**

Hyperuricemia and gout are receiving an increasing scientific and medical attention because of their relatively high prevalence and their association with relevant co-morbidities. Recently, it has been suggested that gout patients have an altered gut microbiota. The first objective of this study was to investigate the potential of some *Ligilactobacillus salivarius* strains to metabolize purine-related metabolites. The second objective was to evaluate the effect of administering a selected potential probiotic strain in individuals with a history of hyperuricemia.

**Methods:**

Inosine, guanosine, hypoxanthine, guanine, xanthine, and uric acid were identified and quantified by high-performance liquid chromatography analysis. The uptake and biotransformation of these compounds by a selection of *L. salivarius* strains were assessed using bacterial whole cells and cell-free extracts, respectively. The efficacy of *L. salivarius* CECT 30632 to prevent gout was assessed in a pilot randomized controlled clinical trial involving 30 patients with hyperuricemia and a history of recurrent gout episodes. Half of the patients consumed *L. salivarius* CECT 30632 (9 log_10_ CFU/day; probiotic group; *n* = 15) for 6 months while the remaining patients consumed allopurinol (100–300 mg/daily; control group; *n* = 15) for the same period. The clinical evolution and medical treatment received by the participants were followed, as well as the changes in several blood biochemical parameters.

**Results:**

L. salivarius CECT 30632 was the most efficient strain for inosine (100%), guanosine (100%) and uric acid (50%) conversion and, therefore, it was selected for the pilot clinical trial. In comparison with the control group, administration of *L. salivarius* CECT 30632 resulted in a significant reduction in the number of gout episodes and in the use of gout-related drugs as well as an improvement in some blood parameters related to oxidative stress, liver damage or metabolic syndrome.

**Conclusion:**

Regular administration of *L. salivarius* CECT 30632 reduced serum urate levels, the number of gout episodes and the pharmacological therapy required to control both hyperuricemia and gout episodes in individuals with a history of hyperuricemia and suffering from repeated episodes of gout.

## Introduction

Purines are part of nucleosides (adenosine and guanosine) from which adenosine monophosphate (AMP) and guanosine monophosphate (GMP) are synthesized and, in turn, used for nucleic acids (DNA/RNA) synthesis. Also, purine derivatives are metabolically relevant molecules involved in cell survival and proliferation, as they act as energy cofactors (ATP, GTP), intracellular signal transduction molecules (cyclic AMP, cyclic GMP), part of coenzymes [nicotinamide adenine dinucleotide (NAD^+^), nicotinamide adenine dinucleotide phosphate (NADP^+^), and coenzyme A] and universal methyl donors (S-adenosylmethionine; Rosemeyer, 2004). Most of the purines present in cells come from the recycling of derivatives of cellular metabolism (the savage pathway), but they can also arise from *de novo* biosynthesis (a process that happens mainly in the liver although small amounts are also produced in the intestine and the vascular endothelium, among other tissues) or from the diet (Pareek et al., 2021). Excess purine nucleosides are removed by breakdown to uric acid involving the sequential action of several enzymes. Adenosine deaminase transforms adenosine into inosine, and the enzyme purine nucleoside phosphorylase converts inosine and guanosine into hypoxanthine and guanine, respectively, which are further transformed to xanthine by xanthine oxidase (acting on hypoxanthine) and guanine deaminase (acting on guanine). In addition, xanthine oxidase, also known as xanthine oxidoreductase, also converts xanthine into uric acid.

Uric acid exists as urate (deprotonated form; pKa = 5.8) at physiological pH (Mandal and Mount, 2015). The normal concentration ranges for urate in human blood are 1.5–6.0 mg/dL in women and 2.5–7.0 mg/dL in men, which are close to saturation levels and are unusually high concentrations compared to other mammalian species due to the lack of uricase, the enzyme responsible of urate degradation (Álvarez-Lario and Macarrón-Vicente, 2010). Urate concentration in the blood depends on the balance between its rate of synthesis in the body, the amount of xanthine of purines from the diet, and on the rate of urate excretion (Lane and Fan, 2015). Approximately, two-thirds of urate elimination takes place in the kidneys, being excreted in the urine, while the remaining one-third occurs in the gastrointestinal tract (Sorensen, 1965; Maesaka and Fishbane, 1998). When there is an imbalance between urate production and excretion, hyperuricemia, defined as an elevated serum urate concentration (>6.0 mg/dL in women and >7.0 mg/dL in men), occurs (Li et al., 2019; George and Minter, 2022). Hyperuricemia increases the risk of precipitation of monosodium urate crystals (solubility limit at 6.8 mg/dL), which can cause gout and urate kidney stones, and also has been associated with the development and severity of many other conditions including chronic renal disease, cardiovascular diseases and metabolic syndrome due to its role in inducing inflammation, endothelial dysfunction, the proliferation of vascular smooth muscle cells and the activation of the renin-angiotensin system (Yanai et al., 2021).

Some risk factors for hyperuricemia and gout development are non-modifiable factors, such as sex (men are at a higher risk than women), age (risk increases with age), race and/or ethnicity, and genetics (due to genetic variants of renal urate transporters), but others are modifiable factors including diet (alcohol consumption, purine-rich foods, fructose-sweetened beverages), medication, and lifestyle (physical activity, body mass index [BMI]) (MacFarlane and Kim, 2014). However, the two main causes for hyperuricemia and gout are, first, a purine-rich diet (seafood, meat, animal offal, alcoholic beverages, and fructose-containing drinks) which induces urate overproduction and, second, a deficient excretion by kidneys and gut.

In the United States, the prevalence rates of hyperuricemia (defined as a serum urate level of >7.0 mg/dL regardless of sex) and gout were 11.9 and 3.9%, respectively, and about one-third of gout patients reported the use of urate-lowering therapy (Chen-Xu et al., 2019). Among non-United States populations, the prevalence of hyperuricemia and gout is higher in Asian than in European populations, although wide variability has been reported due to differences in genetic background and non-genetic factors (Butler et al., 2021).

Most treatments for patients affected by hyperuricemia are based on three strategies: (a) uricostatic drugs inhibiting the production of urate (e.g., allopurinol, febuxostat, and topiroxostat) by modulating the activity of a key enzyme (xanthine oxidase) involved in the production of uric acid; (b) uricosuric drugs that promote the excretion of urate in the kidneys by reducing its reabsorption in the renal tubules (e.g., benzbromarone, probenecid, sulfinpyrazone, and lesinurad); or (c) promoting the transformation of urate to more soluble allantoin and hydrogen peroxide (injectable recombinant uricases, such as pegloticase) but, while showing different effectiviness in reducing serum urate levels, most of them are known to have a myriad of side effects (Strilchuk et al., 2019). Unlike other conditions, dietary restriction alone does not always lead to resolution or improvement of hyperuricemia symptoms. Only a few dietary interventions have been described in the literature that resulted in a small decrease in serum urate levels (Vedder et al., 2019).

In the last decade, a wealth of information has arisen on the important role that the human microbiota plays in health and disease (reviewed in Requena and Velasco, 2021; Afzaal et al., 2022). Because alterations in the human microbiota could play a role in the development of various diseases, modifications of the microbiota (probiotics, prebiotics, antibiotics, and fecal microbiota transference) have been proposed as strategies to prevent and treat some illnesses, including hyperuricemia (Rizzatti et al., 2018; Martín and Langella, 2019; Antushevich, 2020; Fan and Pedersen, 2021; Wang et al., 2022). In fact, gout has been linked to gut bacterial dysbiosis: *Bacteroides caccae* and *Bacteroides xylanisolvens* were enriched in the gut microbiota of patients with clinically diagnosed gout while, simultaneously, *Faecalibacterium prausnitzii* was depleted resulting in reduced butyrate biosynthesis and altered purine degradation in the gut (Guo et al., 2016). More recent studies have confirmed that the profiles of gut microbiota and bacterial metabolites were altered in gout patients, and that they may be partly reversed after urate-lowering treatment with febuxostat (Shao et al., 2017; Chu et al., 2021; Lin et al., 2021). At present, the exact mechanisms relating gut microbiota and purine metabolism are unknown. Modulation of gut microbiota composition using probiotics may be a promising intervention to regulate serum urate levels as it has been shown in animal studies with bacterial strains isolated from fermented foods (Li et al., 2014; Cao et al., 2017; Yamada et al., 2017; Wu et al., 2021). Human vagina and milk have been shown to contain unique microbiotas playing key roles in the initial colonization of the infant gut and, most probably, in the short- and long-term health of the human host (Fernández et al., 2020; France et al., 2022). In the past years, our group has characterized bacterial isolates from both milk and vaginal samples from healthy individuals and some of them were revealed to be good probiotic candidates in clinical trials (Arroyo et al., 2010; Fernández et al., 2016; Cárdenas et al., 2019; Martín et al., 2019; Fernández et al., 2021; Jiménez et al., 2021). Therefore, the objective of this study was, first, to select a potential probiotic strain with the ability to metabolize purine-related metabolites and, second, to evaluate the effect of the administration of this potential probiotic strain on individuals with a history of hyperuricemia in order to evaluate the feasibility of a future multicenter randomized controlled trial.

## Materials and methods

### Bacterial strains and culture conditions

A collection of 13 *Ligilactobacillus salivarius* strains were initially included in this study. Such strains had been previously isolated from human milk or vaginal exudate samples obtained from healthy individuals. Routinely, bacterial cultures were transferred (1.5%, v/v) from frozen stock cultures to de Man, Rogosa and Sharpe (MRS, Oxoid, Basingstoke, United Kingdom) broth and incubated aerobically at 37°C for 24 h. Viable bacteria were quantified by spreading decimal dilutions onto MRS agar (1.5%, w/v) plates incubated aerobically at 37°C for 24 h. The results were expressed as the number of colony-forming units (CFU).

### Evaluation of the uptake of guanosine, inosine, and uric acid by whole bacterial cells

The initial screening of the bacterial collection was carried out by using the method described by Li et al. (2014) with some modifications. In brief, bacterial cells were collected from overnight broth cultures (stationary phase) by centrifugation at 4°C and 19,000 ×*g* for 10 min and washed once with the same volume of cold saline (0.85% NaCl, w/v) solution. Then, the cell pellet was suspended in 0.2 mL of phosphate buffer (100 mM K_3_PO_4_, pH 7) and an aliquot was taken to determine the number of viable cells. 0.8 mL of 1.3 mM solution of either guanosine, inosine, and/or uric acid, sterilized by filtration (0.22 μm pore size; Nalgene 176–0020 nylon), were mixed with 100 or 25 μL of the suspension of washed cells (about 10^10^ CFU of viable cells) and the required volume of the phosphate buffer to reach a final volume of 1.4 mL. When indicated, 0.1 mL of either sterile 4 mM glucose or distilled water were added to the mixture to evaluate the uptake of inosine, guanosine, and uric acid in the presence of glucose. The mixtures were incubated in a water bath at 37°C for 60 min. Afterwards, the mixture was centrifuged as above and 540 μL of the cell-free supernatant were mixed with 60 μL of 0.1 M HClO_4_. Following filtration (0.22 μm pore size; Nalgene 176–0020 nylon) to remove particulate material, the filtrate was kept frozen at −20°C until the determination of the concentration of guanosine, inosine, and uric acid by high-performance liquid chromatography (HPLC) analysis.

### Evaluation of the biotransformation of guanosine, inosine, and uric acid

For this purpose, the same procedure described in the previous section was used but, in this case, cells were substituted by cell-free extracts (CFE). To prepare the CFE, concentrated washed cells suspended in 0.2 mL of cold 0.1 M phosphate buffer (pH 7) were mixed with the same volume of glass beads (0.1 mM bead size; Sigma-Aldrich) and lysed by mechanical disruption in an FP120 FastPrep® Instrument (QbioGene, Irvine, California, United Sates). The mixture was subjected to three processing cycles of 20 s of bead-beating at 5 m s^−1^ followed by 2 min incubation in an ice bath to avoid protein denaturalization. After bead-beating, the tubes were centrifuged at 4°C and 19,000 ×*g* for 10 min to sediment cell debris and glass beads. The supernatant (CFE) was transferred to clean tubes and 25 μL were used instead of whole cell suspensions as described above to determine the transformation of guanosine, inosine, and uric acid.

The protein content in the CFE was determined by the Bradford method using a Coomassie Plus (Bradford) Assay Reagent (Thermo Scientific™) and bovine serum albumin (Sigma-Aldrich) as the standard and the microplate procedure in 96-well plates.

### Identification and quantification of inosine, guanosine, hypoxanthine, guanine, xanthine, and uric acid

Inosine, guanosine, hypoxanthine, guanine, xanthine, and uric acid were quantified by HPLC using the method described by Li et al. (2014), with slight modifications. Analyses were carried out using a Zorbax SB-C18 (5 μm, 4.6 × 250 mM) column connected to an HPLC device (Agilent 1,260 Infinity Quaternary LC) with a diode array detector (Agilent Technologies, Waldbronn, Germany). Working solutions (1.3 mM) of all compounds were prepared in phosphate buffer (K_3_PO_4_, 100 mM, pH 7), cleaned and sterilized by passing the solution through a filter (0.22 μm pore size; Nalgene 176–0020 nylon), and degassed by sonication. The separation of the compounds was achieved by using an isocratic flow (0.5 mL/min) of methanol and 0.1% of acetic acid in Milli-Q water (3:97, v/v). The retention times at 245 nm were 5.00, 4.75, 2.46, 2.36, 2.24, and 2.09 min for guanosine, inosine, xanthine, hypoxanthine, guanine, and uric acid, respectively.

Compound quantification was carried out by developing standard curves built using the corresponding pure compounds (Sigma, Alcobendas, Madrid). The analyses were carried out in triplicate.

### Efficacy of *Ligilactobacillus salivarius* CECT 30632 to prevent gout: A pilot randomized controlled clinical trial

A total of 30 patients participated in the study and were recruited at Centro de Diagnóstico Médico (Madrid, Spain). All of them shared hyperuricemia (>7 mg/dL), a history of recurrent gout episodes (≥3 episodes/year), characterized by acute arthritis and requiring treatment with colchicine despite taking allopurinol (100–300 mg/day) as a preventive measure. The definition of case (gout) was performed following the criteria of the Spanish Society for Rheumatology. Exclusion criteria included antibiotic or probiotic treatment within the previous 2 months or suffering a gout episode at recruitment. Sample size for this trial was calculated accepting an alpha risk of 0.05 and a beta risk of 0.2 in a one-side test to find a 400% increase in the number of individuals without gout episodes during the 6-month period of the trial and anticipating a drop-out rate of 4%. Participants were allocated by simple randomization using a computer-generated list of random numbers prepared by an independent researcher who also prepared the envelopes containing the treatment but with no clinical involvement in the trial. Then, half of the patients (*n* = 15; probiotic group) consumed daily, for 6 months, a sachet containing ~9 log_10_ CFU of *L. salivarius* CECT 30632 while the other half of the patients (*n* = 15; control group) consumed allopurinol (100–300 mg/day) for the same period. Physicians and data analysts were kept blinded to the allocation.

Consumption of drugs used for prevention (allopurinol) or treatment (colchicine, non-steroidal inflammatory drugs [NSAIDs]) of gout was recorded throughout the study. BMI was calculated as weight divided by the square of the height (kg/m^2^). Blood samples were obtained at the beginning (T1) and at the end (T2; 6 months) of the study. Blood samples were extracted at Unilabs (Madrid, Spain). The first 8 mL-fraction was collected into a Na-heparin tube to analyze oxidative stress (OS)-related parameters in plasma, including markers of oxidative stress (advanced oxidation protein products [AOPPs], sulfhydryl [SH] groups, thiobarbituric acid reactive substances [TBARS], malondialdehyde [MDA], 8-isoprostaglandin F2α) and indicators of vascular function and blood pressure modulation (nitric oxide, nitrite, nitrate); a second 4 mL-fraction was used to obtain serum for standard biochemistry (serum urate, triglycerides, total cholesterol, aspartate transaminase [AST or GOT], alanine transaminase [ALT or GPT]). Hematology and biochemical analyses of blood samples were performed by Unilabs. Metabolites related to OS and nitric oxide metabolism end products (NOx) were measured in duplicate as described previously (Codoñer-Franch et al., 2011, 2012).

This study was conducted according to the guidelines laid down in the Declaration of Helsinki and was approved by the Ethics Committee of the Hospital Clínico San Carlos (Madrid, Spain) (protocol: CEIC 20/263-E; date of approval: 01/04/2020, act 4.1/20).

### Statistical analysis

The normality of data distribution was analyzed using the Shapiro–Wilks test. Quantitative variables are presented as mean and 95% confidence interval (CI) or standard deviation (SD). The Student’s t-test was used to compare the means of continuous variables having a normal distribution. The *χ*^2^ test, the Fisher exact test or the Freeman–Halton extension of the Fisher exact probability test for a 2 × 4 contingency table were used to compare percentages. One-way repeated measures ANOVA was used to compare the changes in the mean values of blood parameters during the study. Differences were considered significant when the value of *p* < 0.05. Statistical calculations were performed using Statgraphics Centurion 19 version 19.2.01 (Statgraphics Technologies, Inc., The Plains, VA, United States).

## Results

### Initial screening of *Ligilactobacillus salivarius* strains for nucleoside and uric acid uptake

**Figure 1:**
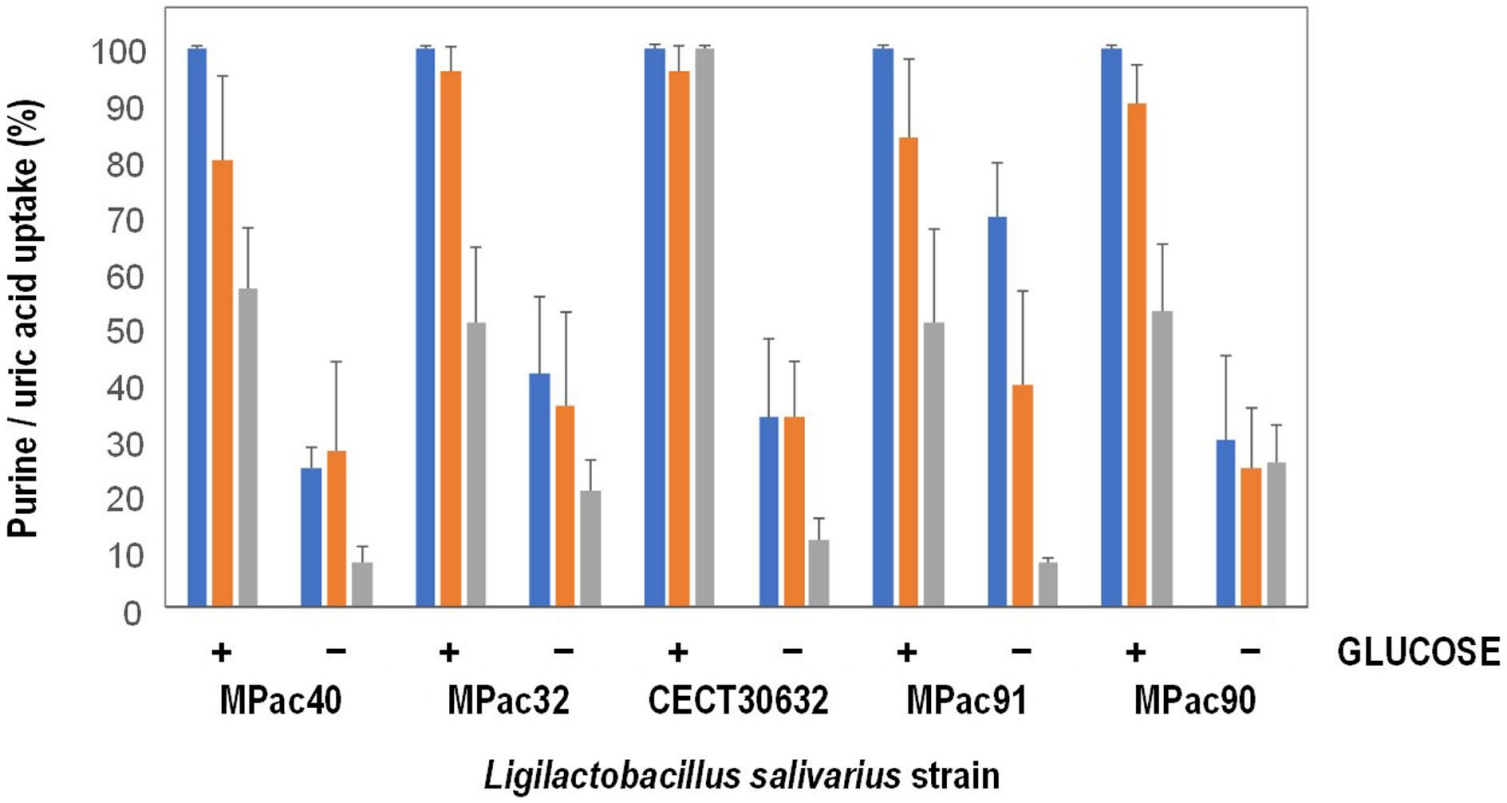
Inosine (blue), guanosine (orange), and uric acid (grey) uptake by whole cells of selected *Ligilactobacillus salivarius* strains in the presence (+) or not (−) of 4 mM glucose.

Most of 13 *L. salivarius* strains included in this study showed a high ability for guanosine and inosine uptake (Supplementary Table S1). The results of uptake capacity of inosine, guanosine, and uric acid in the presence or not of 4 mM glucose of the most active purine-uptaking strains (5 strains) is shown in Figure 1. The five strains transported inosine, guanosine and uric acid into the cytoplasm, although some variability was observed among them. Most of the strains transported lower amounts of uric acid compared to inosine and guanosine, except MPac90 which showed no preference for any of the three compounds. In the presence of glucose, the transport of the three compounds into the cells increased, being *L. salivarius* CECT 30632 the only strain that depleted all the inosine, guanosine, and uric acid (Figure 1).

### Biotransformation of inosine and guanosine by whole cells of *Ligilactobacillus salivarius*

Subsequently, both the transformation of inosine and guanosine inside the cells and the release into the extracellular medium of related metabolites were investigated. The concentrations (as % of transformation) of hypoxanthine, xanthine, and uric acid in the extracellular media when the cells had been incubated in the presence of either inosine or guanosine are shown in Table 1. Guanine was not detected in any sample under these assay conditions.

**Table 1:** Biotransformation of inosine and guanosine by whole cells of selected *Ligilactobacillus salivarius* strains in the presence (+) or not (−) of 4 mM glucose.

			Transformation (%)		
			Product in the extracellular medium		
Strain	Substrate	Glucose (4 mM)	Hypoxanthine	Xanthine	Uric acid
CECT 30632	Inosine	−	97 ± 10	<1	8 ± 3
		+	ND	ND	ND
	Guanosine	−	ND	<1	<1
		+	ND	ND	ND
MPac32	Inosine	−	58 ± 1	<1	19 ± 9
		+	33 ± 11	<1	<1
	Guanosine	−	<1	<1	22 ± 6
		+	<1	<1	<1
MPac40	Inosine	−	89 ± 2	ND	89 ± 1
		+	6 ± 1	ND	<1
	Guanosine	−	ND	ND	2 ± 1
		+	<1	1	1
MPac90	Inosine	−	90 ± 9	2	<1
		+	ND	ND	<1
	Guanosine	−	ND	1	<1
		+	ND	ND	ND
MPac91	Inosine	−	99 ± 9	<1	60 ± 12
		+	83 ± 9	ND	3 ± 2
	Guanosine	−	<1	5 ± 3	99 ± 5
		+	<1	ND	13 ± 4

ND, not detected.

Except for the strain MPac32, inosine was efficiently transformed into hypoxanthine (>89%) and released into the extracellular media by the rest of selected strains (Table 1). When glucose was present in the reaction mixture, the release of hypoxanthine decreased in all the strains, although to a different extent; hypoxanthine was not even detected in the extracellular media of *L. salivarius* CECT 30632 and MPac90. Xanthine was found only in some samples and at a very low concentration (≤ 2% transformation), regardless of the presence of glucose. On the other hand, *L. salivarius* MPac40 and MPac91 secreted also uric acid in addition to hypoxanthine when inosine was supplied in the reaction mixture. *L. salivarius* MPac91 was the most efficient at metabolizing guanosine to uric acid and releasing it from cells (Table 1).

### Biotransformation of inosine and guanosine by cell extracts of *Ligilactobacillus salivarius*

The products obtained after incubation of cell-free intracellular extracts of selected *L. salivarius* strains with inosine and guanosine are shown in Table 2. *L. salivarius* CECT 30632 and MPac32 extracts efficiently transformed inosine and guanosine to hypoxanthine and xanthine (>90%), respectively, which were no further converted to uric acid in a large proportion (<10%). The yield of xanthine from guanosine was lower (<35%) and that of uric acid was higher (>20%) in *L. salivarius* MPac40, MPac90, and MPac91 than in CECT 30632 and MPac32. Most of the inosine present in the reaction mixture containing *L. salivarius* MPac90 extracts was converted to uric acid (>90%) without accumulation of the intermediates hypoxanthine and xanthine, but a lower efficiency of the guanosine transformation was found, yielding 30–45% of xanthine and 8–26% of uric acid. Remarkably, the inclusion of glucose in the reaction mixture did not modify the type and yield of the obtained compounds (Table 2).

**Table 2:** Biotransformation of inosine and guanosine by cell-free extracts of selected *Ligilactobacillus salivarius* strains.

			Transformation (%)		
Strain	Substrate	Glucose (4 mM)	Hypoxanthine	Xanthine	Uric acid
CECT 30632	Inosine	−	92 ± 11	ND	8 ± 6
		+	89 ± 9	ND	8 ± 2
	Guanosine	−	ND	97 ± 7	9
		+	ND	94 ± 8	7 ± 5
MPac32	Inosine	−	92 ± 9	9 ± 7	1
		+	98 ± 8	ND	13 ± 9
	Guanosine	−	ND	89 ± 10	9 ± 5
		+	ND	75 ± 13	7 ± 5
MPac40	Inosine	−	89 ± 24	12 ± 9	30 ± 13
		+	81 ± 1	5 ± 2	18 ± 5
	Guanosine	−	3	33 ± 18	19 ± 9
		+	ND	32 ± 23	ND
MPac90	Inosine	−	10 ± 5	ND	95 ± 11
		+	9 ± 4	ND	93 ± 11
	Guanosine	−	3 ± 1	30 ± 6	8 ± 5
		+	3 ± 1	45 ± 12	26 ± 5
MPac91	Inosine	−	85 ± 18	ND	21 ± 9
		+	100 ± 20	14 ± 9	9
	Guanosine	−	1	25 ± 8	5 ± 3
		+	4 ± 1	35 ± 18	6 ± 3

ND, not detected.

When considering the protein concentration in the reaction mixtures, to somehow relate the extent of transformation to the bacterial enzyme amount, *L. salivarius* CECT 30632 was the most efficient for inosine (100%), guanosine (100%) and uric acid (50%) conversion (Table 3). For this reason, this strain was selected for a pilot clinical trial.

**Table 3:** Extent of inosine, guanosine, and uric acid transformation by cell-free extracts of selected *Ligilactobacillus salivarius* strains.

			Transformation of		
Strain	Protein in CFE* (μg/mL)	Glucose (mM)	Inosine (%)	Guanosine (%)	Uric acid (%)
CECT 30632	11.1 ± 1.20	-	100 ± 12	100 ± 8	51 ± 8
		4	100 ± 12	100 ± 8	51 ± 14
MPac32	16.3 ± 0.48	-	36 ± 26	24 ± 23	51 ± 14
		4	43 ± 25	37 ± 19	51 ± 12
MPac40	8.1 ± 1.57	-	100 ± 12	100 ± 8	14 ± 3
		4	100 ± 12	100 ± 8	13 ± 6
MPac90	26.4 ± 7.32	-	100 ± 12	100 ± 8	4 ± 2
		4	100 ± 12	100 ± 8	6 ± 4
MPac91	14.2 ± 0.44	-	24 ± 18	30 ± 21	51 ± 15
		4	24 ± 22	37 ± 4	53 ± 12

*CFE, cell-free extract.

### Clinical outcomes of the study

This study was conducted between July 2020 and December 2021. A total of 30 volunteers were enrolled and assigned to the probiotic (*n* = 15) or the control (allopurinol) (*n* = 15) groups. There were no withdrawals during the assay and the compliance rate was high (>93%). Baseline characteristics of the participants were comparable, and no significant differences were identified in terms of age, BMI, and the number of gout episodes in the 9 previous months (Table 4).

**Table 4:** Baseline characteristics of the hyperuricemic volunteers (*n* = 30) that participated in this study.

	Control group (*n* = 15)	Probiotic group (*n* = 15)	*p*-value*
	Mean [95% CI] or *n* (%)	Mean [95% CI] or *n* (%)	
Age (years)	54.2 [52.2–56.2]	53.9 [51.9–55.9]	0.802
BMI (kg/m^2^)	32.0 [30.5–33.5]	31.7 [30.2–33.3]	0.784
Gout episodes in the previous 9 months			
0	1 (7)	1 (7)	0.772
1	5 (33)	7 (47)	
2	8 (53)	5 (33)	
3	1 (7)	2 (13)	

*Student’s *t*-tests for age and BMI variables; the Freeman–Halton extension of the Fisher exact probability test for a 2 × 4 contingency table was used to compare the number of gout episodes in the 9 months previous to the study. BMI, body mass index.

**Figure 2:**
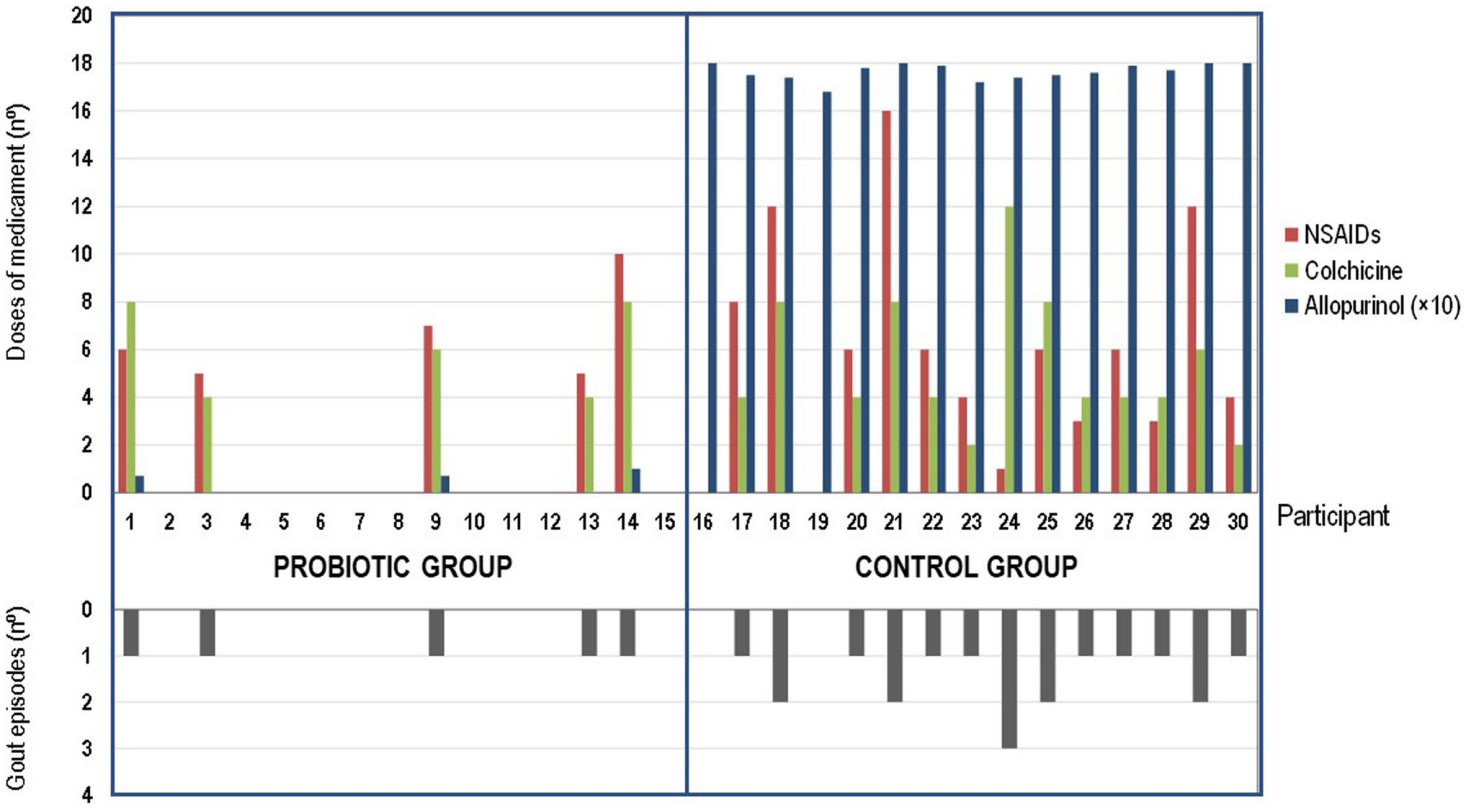
Effect of the probiotic treatment with *Ligilactobacillus salivarius* CECT 30632 on gout-associated symptomatology and use of hyperuricemic-related drugs during the study in the probiotic group (participants 1 to 15) and in the control group (participants 16–30).

The main outcomes of this pilot study are shown in Tables 5, 6 and Figure 2. Daily administration of the probiotic *L. salivarius* CECT 30632 to hyperuricemic individuals was well tolerated and resulted in a significant reduction of the number of gout episodes reported during the period that lasted the study in comparison to the participants that received allopurinol in the control group (the Freeman–Halton extension of the Fisher Exact Probability Test for a 2 × 4 contingency table; *p* = 0.006). In the probiotic group, only 5 out of the 15 volunteers reported a gout episode (only one episode for each of these 5 participants during the trial). In the control group, 13 out of 15 individuals reported gout episodes, and one third of them suffered at least two episodes during the study; the frequency rates reported in the control group during the study were similar to those registered during the 9 months previous to the study (Tables 4, 5).

**Table 5:** Effect of the probiotic treatment with *Ligilactobacillus salivarius* CECT 30632 on the number of gout episodes and the use of hyperuricemic- or gout-related medication during the study.

	Control group (*n* = 15)	Probiotic group (*n* = 15)	*p*-value
	*n* (%)	*n* (%)	
**Number of gout episodes during the study**			
0	2 (13)	10 (67)	0.006^a^
1	8 (53)	5 (33)	
2	4 (27)	-	
3	1 (7)	-	
**Use of allopurinol**			
No	0	12 (80)	<0.001^b^
Yes	15 (100)	3 (20)	
**Use of colchicine**			
No	1 (7)	10 (67)	<0.001^b^
Yes	14 (93)	5 (33)	
**Use of ibuprofen and similar NSAIDs**			
No	2 (13)	10 (67)	0.004^b^
Yes	13 (93)	5 (33)	

^a^The Freeman–Halton extension of the fisher exact probability test for a 2 × 4 contingency table. ^b^Fisher exact probability test (one-tailed). NSAIDs, non-steroidal anti-inflammatory drugs.

**Table 6:** Effect of the probiotic intervention with *Ligilactobacillus salivarius* CECT 30632 on the blood parameters of hyperuricemic participants in the control (*n* = 15) and the probiotic (*n* = 15) groups.

	Control group (*n* = 15)			Probiotic group (*n* = 15)		
Blood parameter	Initial	Final	*p*-value*	Initial	Final	*p*-value*
	Mean (95% CI)	Mean (95% CI)		Mean (95% CI)	Mean (95% CI)	
Uric acid (mg/dL)	9.04 (8.90–9.18)	9.03 (8.89–9.17)	0.922	9.04 (8.72–9.36)	7.90 (7.58–8.22)	<0.001
AOPPs (μmol/L)	61.39 (61.21–61.57)	61.21 (61.03–61.40)	0.163	60.35 (59.41–61.29)	50.84 (49.90–51.78)	<0.001
SH groups (μmol/L)	434.18 (432.96–435.40)	434.17 (432.95–435.40)	0.994	434.28 (432.22–436.34)	432.87 (430.81–434.92)	0.315
TBARS (μmol/L)	26.46 (26.00–26.93)	26.63 (26.17–27.09)	0.583	26.42 (26.11–26.73)	26.06 (25.75–26.37)	0.106
MDA (μmol/L)	2.01 (1.97–2.04)	2.01 (1.98–2.04)	0.872	2.00 (1.97–2.04)	1.89 (1.92–1.97)	<0.001
8–Isoprostaglandin F2α (pg/mL)	184.12 (183.08–185.16)	184.24 (183.02–185.28)	0.863	183.85 (181.74–185.97)	161.03 (158.91–163.14)	<0.001
NOx (μmol/L)	23.58 (23.00–24.17)	23.87 (23.28–24.45)	0.471	23.66 (23.15–24.17)	22.83 (22.31–23.34)	0.027
Nitrite (μmol/L)	2.02 (1.98–2.01)	2.04 (2.00–2.08)	0.637	2.01 (1.98–2.05)	2.01 (1.98–2.04)	0.833
Nitrate (μmol/L)	21.97 (21.51–22.42)	21.88 (21.43–22.34)	0.794	22.11 (21.70–22.53)	21.89 (21.47–22.30)	0.425
Triglycerides (mg/dL)	164.85 (163.52–166.19)	164.75 (163.42–166.09)	0.911	164.47 (161.03–167.92)	134.95 (131.50–138.40)	<0.001
Total cholesterol (mg/dL)	188.86 (187.82–189.91)	188.36 (187.31–189.40)	0.475	189.00 (185.84–193.27)	161.67 (157.95–165.39)	<0.001
GOT (IU/L)	32.93 (32.26–33.60)	33.00 (32.26–33.66)	0.884	32.19 (30.93–33.45)	30.27 (29.01–31.53)	0.037
GPT (IU/L)	37.65 (36.62–38.67)	38.21 (37.19–39.24)	0.413	38.79 (37.47–40.10)	36.66 (35.35–37.98)	0.028

^*^One-way repeated measures ANOVA. AOPPs, advanced oxidation protein products; SH groups, sulfhydryl groups; TBARS, thiobarbituric acid-reactive substances; MDA, malondialdehyde; GOT, glutamic oxoloacetic transaminase; GPT, glutamic pyruvic transaminase; NOx, nitric oxides; IU, international units.

Colchicine and ibuprofen (or related NSAIDs) doses used during the study by the participants of the control (allopurinol) group (70 and 87 total doses, respectively) were much higher than those required by the volunteers of the probiotic group (30 and 33, respectively) (one-way ANOVA; *p* = 0.023 for colchicine and *p* = 0.022 for ibuprofen or related NSAIDs; Figure 2).

There were no differences in the number of doses of either colchicine (*t*-test: *p* = 0.669) or NSAIDs (*t*-test: *p* = 0.964) taken per individual between the individuals in the control group (*n* = 13) or those in the probiotic group (*n* = 5) who suffered gout episodes. Among such participants, the mean (95% CI) of the number of doses per participant was 5.4 (3.6–7.1) doses and 6.7 (4.1–9.3) doses for colchicine or ibuprofen (and related NSAIDs), respectively, in the control group, and 6.0 (3.5–8.5) doses and 6.6 (4.0–9.2) doses for colchicine or ibuprofen (and related NSAIDs), respectively, in the probiotic group. While all the individuals in the control group daily received allopurinol throughout the study, the use of this drug was prescribed for only 5 participants of the probiotic group (Figure 2).

Results of blood analyses performed before the start and at the end of the study to determine the effect of the probiotic intervention in serum urate, markers of oxidative stress (AOPPs, SH groups, TBARS, MDA, 8-isoprostaglandin F2α), indicators of vascular function and blood pressure modulation (NOx, nitrite, nitrate), lipid profile (triglycerides, total cholesterol) and indicators of damage of liver and/or other tissues (AST, ALT) are shown in Table 6. There were no significant changes in the levels of any of the blood parameters tested in the control group individuals. However, relevant changes were observed in the probiotic group in most of the parameters analyzed after the probiotic intervention. Serum urate level was reduced from a mean (95% CI) value of 9.04 (8.72–9.36) mg/dL to 7.90 (7.58–8.22) mg/dL (one-way repeated measures ANOVA; *p* < 0.001). The levels of AOPPs, MDA, and 8-isoprostaglandin F2α were reduced by 16, 6, and 12% (one-way repeated measures ANOVA; *p* < 0.001) in the participants of the probiotic group at the end of the study. In contrast, the levels of SH groups and TBARS did not changed. Serum nitrite and nitrate concentrations did not experience variation but that of NOx decreased by a mean value of 0.83 μmol/L (one-way repeated measures ANOVA; *p* = 0.027). Differences were also found in the mean value of serum triglycerides and total cholesterol that were reduced by 29.52 and 27.88 mg/dL, respectively (one-way repeated measures ANOVA; *p* = 0.000). Individuals in the probiotic group also experienced an improvement in their serum AST and ALT levels since they were significantly reduced by 1.92 and 2.13 IU/L, respectively (one-way repeated measures ANOVA; *p* = 0.037 and *p* = 0.028).

The CONSORT 2010 checklist of information to include when reporting a pilot or feasibility trial^1^ can be found as Supplementary material (Supplementary Table S2; Eldridge et al., 2016).

## Discussion

Gout is a painful arthritis that may occur in hyperuricemic patients and is caused by the precipitation of monosodium urate crystals in joints and soft tissues, which activates the inflammasome with the concomitant release of IL-1βb (Dalbeth et al., 2021). The intestinal tract is the second organ responsible for the excretion of urate (20–30%) and this process is driven by efflux transporters such as BCRP/ABCG2, which in addition to being present in the kidney is also highly expressed at the apical membrane of the small intestine (Hosomi et al., 2012). It has been hypothesized that the intestinal microbiota might contribute to the reduction of the serum urate levels by different mechanisms: promoting catabolism of purines and urate, secreting microbial metabolites that facilitate urate excretion, and alleviating the intestinal inflammation that is associated with hyperuricemia (Wang et al., 2022). Considering that the gut microbiota profile of gout patients is usually altered, probiotics have been proposed as an interesting alternative to treat hyperuricemia and deposition of urate crystals, without the adverse effects of classical pharmacological therapies (Guo et al., 2016). Different lactobacilli strains have been proven to effectively prevent hyperuricemia and/or reduce serum urate levels in human trials (Li et al., 2014; Yamada et al., 2017; Wang et al., 2019). The results of our pilot study show that serum urate levels and, of utmost relevance, the number of gout episodes and the amount of medication used to prevent or treat acute gout episodes were significantly reduced in individuals with hyperuricemia after the consumption of the probiotic strain *L. salivarius* CECT 30632, at a daily dose of 10^9^ UFC for 6 months.

Similarly to Li et al. (2014), in this study, uric acid and two precursors for uric acid generation (guanosine and inosine) were used as substrates to select potential gout-preventing strains. Our working hypothesis was that increased intestinal clearance of uric acid and/or its precursors by potential probiotic bacteria would eventually lead to lower serum urate levels. Our results showed that almost all of the 13 *L. salivarius* strains included in the first screening had high inosine and guanosine uptake capacity. Lactic acid bacteria are ubiquitous in rich ecological niches, such as plants, raw and processed foods, wastewater sludge, and the gastrointestinal tract and other mucosal surfaces of a wide variety of host species (George et al., 2018). The adaptation of many lactic acid bacteria to nutrient-rich environments has resulted in an auxotrophy for purines and pyrimidines (Kilstrup et al., 2005). Dedicated transporters have been described for both nucleosides and purine bases, whereas nucleotides (the first degradation product of nucleic acids) must be dephosphorylated by phosphatases before being transported into the bacterial cell. The nucleoside and/or the purine base, once inside the bacterial cell, enter the salvage pathway for nucleotide synthesis or are degraded to purine metabolites (Kilstrup et al., 2005). Nucleoside transport has been proven in many different species of lactic acid bacteria (Li et al., 2014; Hsieh et al., 2021; Lee et al., 2022), but the incorporation of inosine and guanosine by *L. salivarius* strains of human origin is described for the first time in this study. Additionally, it shows that whole cells of 5 selected *L. salivarius* strains also uptake uric acid, although to a lesser extent than nucleosides. Under the assay conditions, only one strain, *L. salivarius* CECT 30632, incorporated all the inosine, guanosine and uric acid present in the extracellular media when abundant glucose was provided. The availability of an easily metabolizable carbon source (glucose) may increase the transport of adenosine, guanosine and uric acid, which will be subsequently used as a nitrogen source for the bacterial cells.

A more detailed characterization of the fate of the nucleosides inosine and guanosine revealed that inosine was metabolized to hypoxanthine which was, then, excreted outside the cell under glucose restriction conditions but not in a glucose-rich medium. Therefore, the absence of glucose will favor the use of the pentose sugar of the nucleosides as a carbon source for *L. salivarius* CECT 30632, MPac32, and MPac90, as it has been reported in *Lactilactobacillus sakei* CTC 494 (Rimaux et al., 2011). These strains also transported efficiently guanosine, but xanthine was not detected in the extracellular medium, and glucose availability did not modify this result. Uric acid was not detected in the extracellular media following the uptake of inosine and guanosine by *L. salivarius* CECT 30632 and MPac90, but different amounts of this metabolite were detected in the case of *L. salivarius* MPac32, MPac40 and MPac91.

This study also explored the enzymatic potential of the strains to transform inosine, guanosine and uric acid. All the strains, except MPac90, transformed efficiently inosine into hypoxantine. In an animal model, it has been shown that hypoxanthine in the colon modulates energy metabolism (promoting the purine savage pathway) and improves the barrier function in intestinal epithelial cells (Lee et al., 2018). In addition, *L. salivarius* CECT 30632 and MPac32 also metabolized guanosine to xanthine. This transformation in the gut lumen could be advantageous if purine bases are less efficiently transported across the apical membrane of human intestinal epithelial as it has been reported in animal models (Stow and Bronk, 1993). The transformation of inosine and guanosine into hypoxanthine and xanthine suggests that these strains possess purine nucleosidases (Kilstrup et al., 2005). This transformation would be advantageous because some bacteria favor the uptake of purine bases over purine nucleosides to be incorporated into the savage pathway for the synthesis of nucleotides (Yamada et al., 2017).

The administration of *L. salivarius* CECT 30632 for 6 months to individuals with hyperuricemia and a history of acute gout episodes included in this study resulted in a significant reduction (~1 mg/dL) of serum uric acid. This reduction was not enough to reset uric acid concentration to normal reference range values, but it was highly effective in terms of reducing the number of gout attacks. This indicates that the mechanism involved in the reduction of gout episodes by *L. salivarius* CECT 30632 may be related, at least partly, to effects other than the metabolism of nucleosides, purine bases and uric acid. Provision of nutrients to intestinal epithelial cells for energy that promote intestinal uric acid excretion, alleviating inflammation associated with gout and regulation of the expression of uric acid transporters are alternative mechanisms by which intestinal microbiota could reduce hyperuricemia and gout (Yin et al., 2022). Very recently, a study has also described the potential of *Limosilactobacillus fermentum* GR-3 as a therapeutic adjuvant in humans for probiotic treatment of hyperuricemia (Zhao et al., 2022).

The lower requirement of pharmacotherapy is another benefit derived from the administration of the probiotic since adverse events have been linked to most of the current long-term pharmacological strategies to lower serum urate levels, including allopurinol and colchicine (Benn et al., 2018). High doses of allopurinol, a competitive xanthine oxidase inhibitor, can result in severe cutaneous adverse reactions that are associated in some patients with drug-induced liver injury leading to high mortality rates as demonstrated in a 10-year multi-center prospective study (Huang et al., 2021). In patients who experience adverse events related to allopurinol treatment, the alternative is febuxostat (a noncompetitive xanthine oxidase inhibitor), but it has been associated with an increased cardiac risk (White et al., 2018). Colchicine is the most widely used anti-inflammatory drug as a pharmacologic approach to acute gout and is associated with gastrointestinal adverse effects (diarrhea, nausea, vomiting, cramps, and pain) (Qaseem et al., 2017).

In addition, the intake of *L. salivarius* CECT 30632 resulted in a significant decrease in the blood levels of some markers of oxidative stress (AOPPs, MDA and 8-isoprostaglandin F2α), nitric oxide, triglycerides, total cholesterol, GOT and GPT. Decreases in such parameters are relevant because of the close association between gout and metabolic syndrome (Raya-Cano et al., 2022). Gout is an increasingly relevant condition because of its relatively high prevalence, its impact on well-being and healthcare costs and, most importantly, its association with important co-morbidities that are usually considered as manifestations of the metabolic syndrome including hypertension, hyperlipidemia, cardiovascular disease, liver disease, renal disease, type 2 diabetes, and obesity (Thottam et al., 2017; Kimura et al., 2021). Interestingly, the administration of another *L. salivarius* strain for lactational mastitis (another inflammatory condition) also led to a significant reduction in the same blood parameters (Espinosa-Martos et al., 2016). Other studies have shown that different *L. salivarius* strains are able to reduce the blood levels of total cholesterol, low-density lipoprotein (LDL) cholesterol, and triglycerides in broiler chicken (Shokryazdan et al., 2017) or healthy young humans (Rajkumar et al., 2015), or to attenuate their normal pregnancy-induced rise in women with gestational diabetes, who are at risk of future metabolic syndrome (Lindsay et al., 2015). Chuang et al. (2016) evaluated whether heat-killed cells obtained from either a *L. salivarius* or a *Lactobacillus johnsonii* strain were able to prevent alcoholic liver damage in rats after acute alcohol exposure and found that only those obtained from the *L. salivarius* strain reduced serum AST and triglyceride levels.

The clinical study presented in this work was an initial pilot trial and, therefore, it has the inherent limitation of the relatively low number of individuals included and, as consequence, the results must be confirmed in future multicenter randomized placebo-controlled trials. Moreover, the treatment was limited to 6 months and the analyses were performed immediately at its end, with no follow-up of participants to check the endurance of the observed positive effects. However, this pilot trial has revealed the high potential of *L. salivarius* CECT 30632 to reduce the burden associated with hyperuricemia and gout. Measured parameters in this study to assess the potential benefits of the strain for gout prevention were related to clinical or metabolic outcomes (gout episodes, gout-related medication, and standard blood analyses). Such parameters are more appealing or understandable for medical purposes that changes in the fecal microbiome, whose interpretation is frequently very difficult or even impossible for medical practitioners. In future trials, however, it should be evaluated whether changes in the fecal microbiome occur, since the alteration of the intestinal microbiome balance may favor multiple changes in gastrointestinal physiology and inflammatory status. This knowledge may reveal the involvement of specific microorganisms or consortia in the onset and/or progression of hyperuricemia, improve the diagnosis and treatment of this pathology, and help to develop more effective therapeutic strategies by manipulating the gut microbiota, including the selection of probiotics than are even more effective.

In conclusion, in this study, it was shown that the regular administration of *L. salivarius* CECT 30632 did reduce serum urate levels, the number of gout episodes and, the pharmacological therapy required to control both the hyperuricemia and the gout episodes involving individuals with a history of hyperuricemia and suffering repeated gout episodes.

## Data availability statement

The raw data supporting the conclusions of this article will be made available by the authors, without undue reservation.

## Ethics statement

The studies involving human participants were reviewed and approved by The Ethics Committee of the Hospital Clínico San Carlos (Madrid, Spain). The patients/participants provided their written informed consent to participate in this study.

## Author contributions

LF and JR designed and coordinated the study. DB directed the recruitment of participants and the diagnosis of gout episodes. MG, JS, and BO processed the samples and performed the *in vitro* assays. CA and LF performed the statistical analysis. LF, BO, and JR drafted the manuscript. All authors contributed to the article and approved the submitted version.

## Funding

This work was funded by a grant conceded by the Complutense University of Madrid (Spain) to the research group.

## Conflict of interest

The authors declare that the research was conducted in the absence of any commercial or financial relationships that could be construed as a potential conflict of interest.

## Publisher’s note

All claims expressed in this article are solely those of the authors and do not necessarily represent those of their affiliated organizations, or those of the publisher, the editors and the reviewers. Any product that may be evaluated in this article, or claim that may be made by its manufacturer, is not guaranteed or endorsed by the publisher.

## Footnotes

^1^ www.consort-statement.org
