# Supplementary material for: A randomized pilot trial assessing the reduction of gout episodes in hyperuricemic patients by oral administration of Ligilactobacillus salivarius CECT 30632, a strain with the ability to degrade purines
Source: Front Microbiol. 2023 Feb 14;14:1111652. doi: 10.3389/fmicb.2023.1111652 (PMC9971985; doi:10.3389/fmicb.2023.1111652)
Supplement: Supplementary file 1 [file Data_Sheet_1.PDF]

Supplementary Material

A randomized pilot trial assessing the reduction of gout episodes in hyperuricemic patients by oral administration of *Ligilactobacillus salivarius* CECT 30632, a strain with the ability to degrade purines

Juan M. Rodríguez[1], Marco Garranzo[2], José Segura[2], Belén Orgaz[2], Rebeca Arroyo[1], Claudio Alba[1], David Beltrán[3], Leónides Fernández[2,*]

[1]Dpt. Nutrition and Food Science, Complutense University of Madrid, Madrid, Spain

[2]Dpt. Galenic Pharmacy and Food Technology, Complutense University of Madrid, Madrid, Spain

[3]Centro de Diagnóstico Médico, Ayuntamiento de Madrid, Madrid, Spain

*** Correspondence:**

leonides@cm.es (L. Fernández), jmrodrig@ucm.es (J.M. Rodríguez)

1 Supplementary Tables

Supplementary Table S1. Initial screening of *Ligilactobacillus salivarius* strains for inosine and guanosine uptake using whole cells.

Strain	Nucleoside uptake (%)[1]	
	Inosine	Guanosine
MPac32	100 ± 12	100 ± 8
MPac33	100 ± 12	97 ± 1
MPac35	100 ± 12	91 ± 7
MPac36	83 ± 22	76 ± 14
MPac40	100 ± 12	100 ± 8
MPac45	100 ± 12	96 ± 2
MPac53	99 ± 13	95 ± 3
MPac58	88 ± 15	82 ± 10
CECT30632	100 ± 12	97 ± 1
MPac61	100 ± 15	96 ± 13
MPac88	100 ± 12	92 ± 8
MPac90	100 ± 12	100 ± 8
MPac91	100 ± 12	97 ± 1

[1]Mean ± standard deviation. 100% meaning that the reactive was not detected.

Supplementary Table S2. CONSORT 2010 checklist of information to include when reporting a pilot or feasibility trial*

Section/Topic	Item No	Checklist item	Reported on page No
**Title and abstract**			
	1a	Identification as a pilot or feasibility randomised trial in the title	1, line 1
	1b	Structured summary of pilot trial design, methods, results, and conclusions (for specific guidance see CONSORT abstract extension for pilot trials)	1, lines 25-31
**Introduction**			
Background and objectives	2a	Scientific background and explanation of rationale for future definitive trial, and reasons for randomised pilot trial	2, lines 81-108
	2b	Specific objectives or research questions for pilot trial	2, lines 114-118
**Methods**			
Trial design	3a	Description of pilot trial design (such as parallel, factorial) including allocation ratio	5, lines 173-173, 180-183
	3b	Important changes to methods after pilot trial commencement (such as eligibility criteria), with reasons	Not applicable
Participants	4a	Eligibility criteria for participants	5, lines 176-179
	4b	Settings and locations where the data were collected	5, line 176
	4c	How participants were identified and consented	5, lines 198-200
Interventions	5	The interventions for each group with sufficient details to allow replication, including how and when they were actually administered	5. lines 182-184
Outcomes	6a	Completely defined prespecified assessments or measurements to address each pilot trial objective specified in 2b, including how and when they were assessed	5, lines 185-197

	6b	Any changes to pilot trial assessments or measurements after the pilot trial commenced, with reasons	Not applicable
	6c	If applicable, prespecified criteria used to judge whether, or how, to proceed with future definitive trial	Not applicable
Sample size	7a	Rationale for numbers in the pilot trial	5, lines 181-184
	7b	When applicable, explanation of any interim analyses and stopping guidelines	Not applicable
Randomisation:			
Sequence generation	8a	Method used to generate the random allocation sequence	5. lines 184-185
	8b	Type of randomisation(s); details of any restriction (such as blocking and block size)	
Allocation concealment mechanism	9	Mechanism used to implement the random allocation sequence (such as sequentially numbered containers), describing any steps taken to conceal the sequence until interventions were assigned	5. lines 184-185
Implementation	10	Who generated the random allocation sequence, who enrolled participants, and who assigned participants to interventions	5. lines 185-186
Blinding	11a	If done, who was blinded after assignment to interventions (for example, participants, care providers, those assessing outcomes) and how	5. lines 189-190
	11b	If relevant, description of the similarity of interventions	Not applicable
Statistical methods	12	Methods used to address each pilot trial objective whether qualitative or quantitative	5, lines 208-214, and 6, lines 215-216
**Results**			
Participant flow (a diagram is strongly recommended)	13a	For each group, the numbers of participants who were approached and/or assessed for eligibility, randomly assigned, received intended treatment, and were assessed for each objective	7, lines 263-265

	13b	For each group, losses and exclusions after randomisation, together with reasons	
Recruitment	14a	Dates defining the periods of recruitment and follow-up	5, line 175
	14b	Why the pilot trial ended or was stopped	Not applicable
Baseline data	15	A table showing baseline demographic and clinical characteristics for each group	20
Numbers analysed	16	For each objective, number of participants (denominator) included in each analysis. If relevant, these numbers should be by randomised group	21-22, Figure 2
Outcomes and estimation	17	For each objective, results including expressions of uncertainty (such as 95% confidence interval) for any estimates. If relevant, these results should be by randomised group	21-22, Figure 2
Ancillary analyses	18	Results of any other analyses performed that could be used to inform the future definitive trial	22
Harms	19	All important harms or unintended effects in each group (for specific guidance see CONSORT for harms)	7, lines 269-270
	19a	If relevant, other important unintended consequences	7, lines 302-308
**Discussion**			
Limitations	20	Pilot trial limitations, addressing sources of potential bias and remaining uncertainty about feasibility	10, lines 404-406
Generalisability	21	Generalisability (applicability) of pilot trial methods and findings to future definitive trial and other studies	10, lines 407-408
Interpretation	22	Interpretation consistent with pilot trial objectives and findings, balancing potential benefits and harms, and considering other relevant evidence	10, lines 408-412
	22a	Implications for progression from pilot to future definitive trial, including any proposed amendments	10, lines 412-413
**Other information**			

Registration	23	Registration number for pilot trial and name of trial registry	Not applicable
Protocol	24	Where the pilot trial protocol can be accessed, if available	Not applicable
Funding	25	Sources of funding and other support (such as supply of drugs), role of funders	10, lines 429-430
	26	Ethical approval or approval by research review committee, confirmed with reference number	10, lines 419-420

*Eldridge, S.M., Chan, C.L., Campbell, M.J., Bond, C.M., Hopewell, S., Thabane, L., et al. (2016). CONSORT 2010 statement: extension to randomised pilot and feasibility trials. *BMJ*. 355, i5239. doi: 10.1136/bmj.i5239
